# Predictive Factors Associated With the Utilization of Vocational Rehabilitation for Occupational Diseases in Finland: A Multiregister Analysis

**DOI:** 10.1097/JOM.0000000000003435

**Published:** 2025-05-28

**Authors:** Ville Ojanen, Mikko Korhonen, Kirsi Koskela, Tiia Reho, Riitta Sauni

**Affiliations:** From the Tampere University, Faculty of Medicine and Health Technology, Tampere, Finland (V.O., M.K., K.K., T.R., R.S.); Finnish Institute of Occupational Health, Tampere, Finland (V.O., K.K.); and Varma Mutual Pension Insurance Company, Helsinki, Finland (T.R.).

**Keywords:** occupational disease, rehabilitation, vocational, asthma, dermatitis, rhinitis

## Abstract

**Objectives:**

The aims of the study were to evaluate the utilization of vocational rehabilitation (VR) in occupational diseases (ODs) and to determine associated predictive factors.

**Methods:**

The national register data of recognized ODs in Finland (2005–2018) were combined with insurance compensation data and sociodemographic data. We analyzed the effect of the diagnosis, age group, sex, socioeconomic group, and number of allergic ODs on VR utilization.

**Results:**

Of the 24,237 working aged subjects with a recognized OD, 1615 (6.7%) had received VR. Allergic ODs of the skin or respiratory system accounted for 76% of VR cases. The probability of VR decreased significantly with age.

**Conclusions:**

Utilization of VR varied substantially between ODs and age groups. It is important to assess the need for VR services when the OD is diagnosed, especially in the case of work-related hypersensitivity.

## Abstract 2

This study highlights that work-related allergies are the most common reason for vocational rehabilitation among occupational diseases. Patients with occupational sensitization often require complete cessation of exposure and need support to transition to new occupations. Such support can mitigate the socioeconomic impact of their condition.

## Graphical abstract

**Figure:**
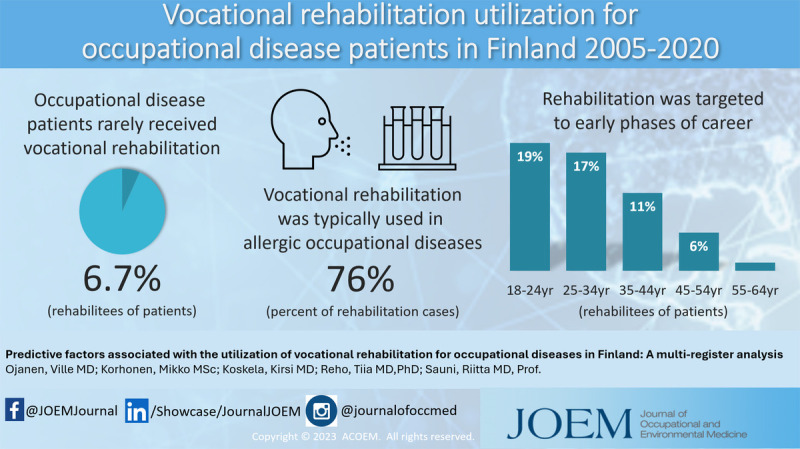

**LEARNING OUTCOMES**

- Vocational rehabilitation was most often utilized in allergic occupational diseases to promote work ability and employability.
- Subjects younger than 35 years of age were most likely to receive vocational rehabilitation.
- Manufacturing and agriculture were the most prominent industry sectors regarding the vocational rehabilitation of occupational diseases.

Occupational diseases (OD) have a substantial effect on health and the economy. The estimate of work-attributed disability-adjusted life-years in 2019 was over 180 million and the total economic loss was 6% of the global gross domestic product. Occupational injuries covered only 13% of losses, thus the majority was linked to other work-related diseases.^1^

OD may end one’s career prematurely or affect one’s ability to stay in the labor market.^2–5^ Work disability caused by an OD may be job-specific; thus, it may be overcome with a change of tasks, work, or profession. Especially when workplace adjustments are insufficient for the cessation or reduction of the exposure, vocational rehabilitation (VR) can be used to promote employment after an OD diagnosis.

Previous studies on VR utilization in ODs appear to be scarce both in Finland and internationally. A Croatian study found that about 5% of subjects with various ODs had received VR, while more than one third of the subjects had received a disability pension.^6^ A Finnish follow-up study and a Canadian return-to-work study on occupational hand eczema revealed that about one fifth of the subjects had been retrained because of their OD.^7,8^ Another Finnish study on occupational rhinitis patients revealed that 17% were re-educated because of the OD.^9^ A short-term follow-up study on Finnish occupational asthma patients found 11% of them in retraining 6 months after diagnosis.^10^ Recent results from a Finnish long-term follow-up study revealed that 36% of occupational asthma patients had received VR due to their OD.^11^ These studies had a limited population and/or focus on a specific OD; consequently, they were unable to provide a comprehensive view of VR utilization in ODs.

The aim of this study was to investigate the overall utilization of VR in ODs; its distribution between different OD diagnoses, industry fields, and occupations; and the determination of possible predictive factors for VR.

## METHODS

We designed an observational cohort study on the VR of working-aged patients with diagnosed ODs in Finland. The study population was formed based on recognized cases of ODs registered in the Finnish Register of Occupational Diseases (FROD) between 2005 and 2018.

### Finnish Setting

OD is defined by the Workers’ Compensation Act in Finland as an illness that is likely to be primarily caused by a physical, chemical, or biological factor affecting the work. Repetitive strain injuries are recognized as an OD caused by a physical agent if the work includes repetitive motions that strain the upper extremity.^12^ A list of common ODs together with their typical causes are included in Finnish legislation, but the list is not exclusive of other diseases. Illnesses caused by psychological factors are not covered as ODs in Finland. The employer is required to provide Workers Compensation Insurance (WCI) for all employees, while it is voluntary for self-employed entrepreneurs. OD can only be recognized for insured individuals, thus excluding uninsured entrepreneurs.

Occupational diseases are registered in the FROD, which is maintained by the Finnish Institute of Occupational Health. The FROD includes all recognized cases in Finland. Approximately 700–800 cases of ODs are recognized in working-aged employees in Finland every year. While the incidence of recognized ODs had diminished in Finland until recently, occupational COVID-19 cases during the pandemic caused a remarkable increase beginning in 2020.^13^

According to legislation, compensable VR in Finland consists of one or several of the following interventions: investigations to establish the need and opportunities for rehabilitation, job coaching in the former or new work, work or training trials, continuing education or re-education, support for starting or modifying entrepreneurial activities, and support for acquiring a vehicle for commuting. In addition, the time committed to rehabilitation and the waiting time involved in the process are covered with a rehabilitation allowance by the WCI.^12^

According to the Workers’ Compensation Act, the WCI is the primary source of compensation for ODs, including VR when considered necessary.^12^ The earnings-related pension and social insurance schemes also provide VR in Finland, but these are considered secondary compensation systems in OD cases, and the criteria for eligibility are different from the WCI. In this study, we present the data focusing only on the WCI as the primary compensation mechanism.

### Data Management

Data on recognized ODs were gathered from the FROD. Each FROD case included information on the case registration year, recognition date, age at recognition, sex, up to three ICD-10 diagnoses, up to three exposure agents linked to the case, the employee’s occupation, and industry sector. Dermatological diagnoses (ICD-10 class L) had additional data to differentiate between different reaction types. Diagnoses were registered in descending order of severity, and the first diagnosis was used in the analysis. The majority of FROD cases (93.5%) included only one diagnosis. The FROD data were obtained from Findata, the Finnish Social and Health Data Permit Authority (data permit THL/3512/14.02.00/2021).

Individual OD compensation data were received from the Finnish Workers’ Compensation Center or Farmers’ Social Insurance Institution Mela. The data included compensation provided to the study population by the WCI between 2005 and 2020. Data on VR expenses and rehabilitation allowance were provided for analysis without description of the content of rehabilitation, including only the amount of expense in euros together with the date and duration of expense.

**FIGURE 1:**
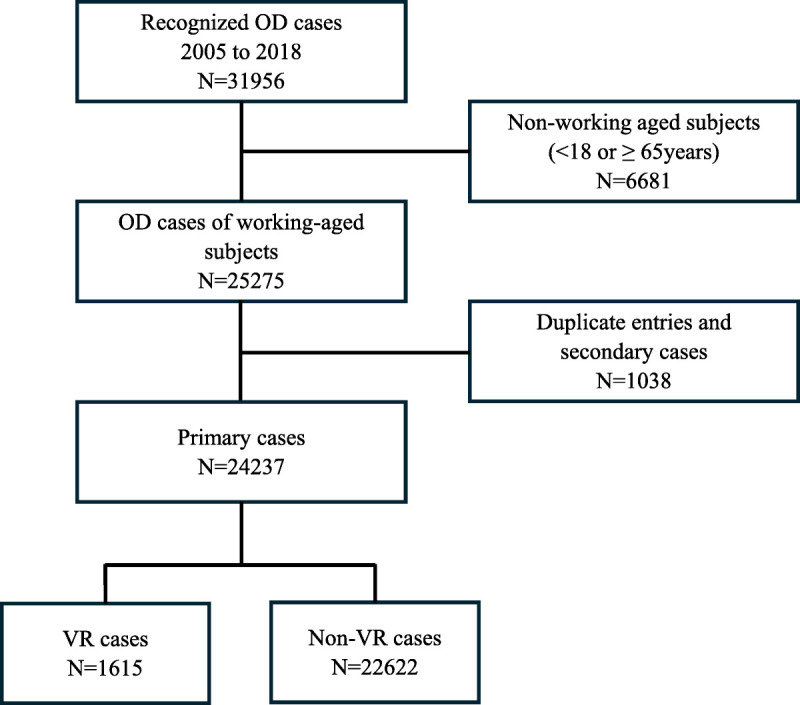
Flow chart of the study population. OD, occupational disease; VR, vocational rehabilitation.

Recognized ODs of the working-age population (18–64 years of age at the time of recognition of OD) between 2005 and 2018 were analyzed in this study. If the same subject had multiple OD cases registered in the FROD, the primary case was the one for which VR compensation was paid, or for the most severe diagnosis in the non-VR group. OD severity was determined through expert opinion by the persistence of the disease and the severity of medical consequences by the corresponding author. After the determination of the primary cases, the subjects’ other FROD cases (secondary cases) were excluded from the analysis to avoid the same subject being included in both the VR and non-VR groups simultaneously. The data exclusion steps are visualized in Figure 1.

Utilization of VR was dichotomized for each case. Follow-up time began in the year the OD was recognized and continued until the subject turned 65, died, or until 2020, the final year of data collection. For the analysis, the diagnostic groups were determined by the primary diagnosis registered for the case. Occupational asthma included recognized cases with ICD-10 diagnoses J45 and J68.3 (irritant-induced occupational asthma). Occupational rhinitis included J30 and J31 diagnoses. Occupational skin diseases included all ICD-10 class “L” diagnoses, but these were subsequently divided into the subgroups of allergic contact dermatitis (ACD), irritant contact dermatitis (ICD), protein contact dermatitis or contact urticaria (PDC/CU), and “Other” according to the additional information obtained from the FROD. Repetitive strain injuries consisted of registered musculoskeletal “M” diagnoses together with upper limb mononeuropathies (G56). The hand-arm vibration syndrome class consisted of Raynaud’s disease (I73.0), effects of vibration (T75.2), and exposure to vibration (Z57.7). Hypersensitivity pneumonitis (J67), toxic encephalopathy (G92), and focal dystonia (G24) were determined by their unique diagnosis code. The remaining ODs were categorized as “Other.”

Further examination of multiple allergic ODs was carried out by combining information on recognized allergic ODs from both primary and secondary cases. Allergic asthma, rhinitis, conjunctivitis, PCD/CU, and ACD were included in the analysis of allergic ODs, whereas irritant-induced asthma and moisture damage-induced asthma were determined as nonallergic ODs in these analyses.

To adjust for confounding factors, Statistics Finland provided individual-level sociodemographic data for the study population. The dataset included sex, age, and socioeconomic group. We examined socioeconomic group divided into manual, lower nonmanual, upper nonmanual, farmer or forestry entrepreneur, other entrepreneur, students, and others. Statistics Finland provided annual data on subjects' educational level and the year of the highest degree. The change in the year or level of the highest degree during VR or in the subsequent year was analyzed in comparison to the situation prior to VR. The duration of VR in years was calculated by identifying all calendar years with recorded VR expenses. Subjects with ongoing VR in 2020 were excluded from these analyses because the end of VR could not be reliably determined. The delay in VR provision was analyzed comparing the recognition year of OD and the year of the first recorded VR expenses. Subjects with unknown recognition date were excluded from this analysis.

For the incidence calculations, national working age labor force data were obtained from Statistics Finland for both occupations (International Standard Classification of Occupations 2010, ISCO)^14^ (available 2010–2018) and field of industry (International Standard Industrial Classification of All Economic Activities revision 4, ISIC)^15^ (available 2007–2018). Code-specific mean workforce was used for the incidence calculation.

Exposure agents were analyzed according to exposure classification by the Finnish Institute of Occupational Health.

For all datasets, personal identity codes and accident ID numbers were pseudonymized by Statistics Finland before releasing the data for analysis. Data management and analysis were conducted through remote access in a data-secured environment provided by Statistics Finland. Only members of the research team could process and analyze the data. Ethics approval was not required for a retrospective registry study with pseudonymized data. The Finnish Social and Health Data Permit Authority Findata was responsible for ensuring the anonymity of the final results.

Data gathered from different sources were combined for analysis using pseudonymized personal identity codes. Insurance data from the Finnish Workers’ Compensation Center and the Farmers’ Social Insurance Institution Mela were paired with the FROD cases by using the pseudonymized accident ID number when available or manually by using ICD-10 diagnosis codes and dates of recognition and registration.

### Statistical Analyses

The STROBE guidelines for cohort studies were followed in manuscript preparation (Document, SDC 1, STROBE checklist, http://links.lww.com/JOM/B953).^16^ The characteristics of VR recipients and nonrecipients were compared at the baseline with chi-squared tests (proportions), and the age variable’s group means with the Mann-Whitney *U* test due to the non-Gaussian distribution. Statistical significance was set at *P* < 0.05.

We estimated odds ratios (OR) for VR utilization with a binary logistic regression model to investigate the predictors of VR. The analysis was performed with diagnostic groups (12 groups, repetitive strain injuries as the reference), age groups (5 groups, youngest as the reference), sex (male as the reference), and socioeconomic group (7 groups, manual workers as the reference) mutually adjusted for.

Another binary logistic regression analysis was performed on allergic ODs to investigate the effect of multiple allergic manifestations on VR utilization. Subjects with a single allergic OD were used as the reference and compared with subjects with multiple allergic ODs and subjects without allergic OD. The regression model was adjusted for age group, sex, and socioeconomic group. Regression analyses and comparison of characteristics were performed using R Statistical Software (v4.0.5; R Core Team 2021).

The incidence rates of VR utilization by occupations and field of industry were calculated per 10,000 person years in the field/occupation. Poisson 95% confidence intervals were calculated for incidence rates using MedCalc Software Ltd. Confidence interval for a rate (Version 23).

## RESULTS

The study population included 24,237 working-aged subjects with a recognized OD. Altogether, 1615 subjects (6.7% of the study population) had received VR because of their OD during follow-up. The mean follow-up time was 9.32 years (SD 4.03) for VR cases and 9.77 years (SD 3.93) for non-VR cases. Table 1 shows the characteristics of VR recipients and nonrecipients.

**TABLE 1:** Characteristics of the Study Population, Stratified by VR of Occupational Disease (*N* = 24,237)

	VR Cases		Non-VR Cases		
	*n* (%)	Mean (SD)	*n* (%)	Mean (SD)	P*
Age		37.65 (10.66)		49.28 (11.63)	<0.001
Age group					
18–24	199 (12.3)		872 (3.9)		<0.001
25–34	501 (31.0)		2,413 (10.7)		
35–44	437 (27.1)		3,424 (15.1)		
45–54	361 (22.4)		6,155 (27.2)		
55–64	117 (7.2)		9,758 (43.1)		
Sex					
Male	786 (48.7)		16,618 (73.5)		<0.001
Female	829 (51.3)		6,004 (26.5)		
Socioeconomic group					
Farmers and forestry entrepreneurs	69 (4.3)		2,191 (9.7)		<0.001
Self-employed persons†	137 (8.5)		1,072 (4.7)		
Upper nonmanual workers	72 (4.5)		1,192 (5.3)		
Lower nonmanual workers	208 (12.9)		3,454 (15.3)		
Manual workers	965 (59.8)		12,295 (54.3)		
Students	58 (3.6)		174 (0.8)		
Other‡	106 (6.6)		2,244 (9.9)		
Occupational disease					
Allergic contact dermatitis	442 (27.4)		1,627 (7.2)		<0.001
Asthma	449 (27.8)		1,036 (4.6)		
Focal dystonia	10 (0.6)		4 (0.0)		
Hand-arm vibration syndrome	40 (2.5)		330 (1.5)		
Hypersensitivity pneumonitis	30 (1.9)		289 (1.3)		
Irritant contact dermatitis	130 (8.0)		1,950 (8.6)		
Other dermatitis	56 (3.5)		368 (1.6)		
Protein contact dermatitis or contact urticaria	125 (7.7)		346 (1.5)		
Repetitive strain injury	60 (3.7)		3,408 (15.1)		
Rhinitis	211 (13.1)		590 (2.6)		
Solvent-induced encephalopathy	14 (0.9)		65 (0.3)		
Other occupational disease	48 (3.0)		12,609 (55.7)		
Total	1,615 (100.0)		22,622 (100.0)		

*Age means: Mann-Whitney *U* test, other variables: chi-squared test.

†Not in agriculture or forestry.

‡Pensioners, unemployed, unknown.

VR, vocational rehabilitation.

Table 2 shows the probability of VR according to OD, sex, age, and socioeconomic group. The majority of VR cases were linked to allergic skin or respiratory system ODs: asthma, rhinitis, PCD/CU, and ACD accounted for 76% of all VR cases. VR was most often used for occupational asthma (OR 45.59; 95% CI 34.11–60.93). Additionally, rhinitis (OR 25.71, 95% CI 18.86–35.03), PCD/CU (OR 30.45, 95% CI 21.55–43.02), and ACD (OR 15.98, 95% CI 12.08–21.13) were significant predictors of VR. Of the nonallergic ODs, the probability of VR was elevated in solvent-induced encephalopathy (OR 25.68; 95% CI 13.23–49.83), and hypersensitivity pneumonitis (OR 23.46; 95% CI 14.37–38.31). Focal dystonia had a very high OR (579.54, 95% CI 170.10–2099.05) for VR, but the number of cases was relatively small.

**TABLE 2:** Predictors of VR Utilization for Occupational Diseases. Binary Logistic Regression Model (*N* = 24,237)

	*n*	VR Cases (%)	Crude OR (95% CI)	Adjusted OR (95% CI)†
Diagnosis				
Repetitive strain injury	3,468	60 (1.7)	1.00	1.00
Irritant contact dermatitis	2,080	130 (6.3)	3.79 (2.77–5.17)	3.86 (2.81–5.29)
Other dermatitis	424	56 (13.2)	8.66 (5.91–12.63)	9.22 (6.26–13.58)
Hand-arm vibration syndrome	370	40 (10.8)	6.88 (4.54–10.43)	10.15 (6.62–15.57)
Allergic contact dermatitis	2,069	442 (21.4)	15.43 (11.71–20.34)	15.98 (12.08–21.13)
Hypersensitivity pneumonitis	319	30 (9.4)	5.90 (3.74–9.29)	23.46 (14.37–38.31)
Solvent-induced encephalopathy	79	14 (17.7)	12.23 (6.51–23.00)	25.68 (13.23–49.83)
Rhinitis	801	211 (26.3)	20.31 (15.05–27.41)	25.71 (18.86–35.03)
Protein contact dermatitis or contact urticaria	471	125 (26.5)	20.52 (14.80–28.46)	30.45 (21.55–43.02)
Asthma	1,492	449 (30.1)	24.45 (18.51–32.29)	45.59 (34.11–60.93)
Focal dystonia	14	10 (71.4)	142.00 (43.31–465.50)	597.53 (170.10–2099.05)
Other occupational disease	12,650	48 (0.4)	0.22 (0.15–0.32)	0.47 (0.32–0.70)
Sex				
Male	17,404	786 (4.5)	1.00	1.00
Female	6,833	829 (12.1)	2.92 (2.64–3.23)	1.11 (0.98–1.26)
Age group				
18–24	1,071	199 (18.6)	1.00	1.00
25–34	2,914	501 (17.2)	0.91 (0.76–1.09)	1.05 (0.85–1.29)
35–44	3,861	437 (11.3)	0.56 (0.47–0.67)	0.81 (0.65–1.00)
45–54	6,516	361 (5.5)	0.26 (0.21–0.31)	0.53 (0.42–0.66)
55–64	9,875	117 (1.2)	0.05 (0.04–0.07)	0.17 (0.13–0.22)
Socioeconomic group				
Manual workers	12,295	965 (7.8)	1.00	1.00
Farmers and forestry entrepreneurs	2,191	69 (3.1)	0.40 (0.31–0.51)	0.14 (0.11–0.19)
Upper nonmanual workers	1,192	72 (6.0)	0.77 (0.60–0.99)	0.31 (0.23–0.42)
Lower nonmanual workers	3,454	208 (6.0)	0.77 (0.66–0.90)	0.39 (0.32–0.46)
Self-employed persons‡	1,072	137 (12.8)	1.63 (1.35–1.97)	1.01 (0.81–1.26)
Students	174	58 (33.3)	4.25 (3.13–5.76)	1.71 (1.18–2.47)
Other (pensioners, unemployed, unknown)	2,244	106 (4.7)	0.60 (0.49–0.74)	1.27 (0.99–1.63)

‡Diagnosis, sex, age group, and socioeconomic group were mutually adjusted for in the model.

‡Not in agriculture or forestry.

CI, confidence interval; OR, odds ratio; VR, vocational rehabilitation.

Men had more ODs than women, but women had more VR cases compared to men. Table 2 shows that 12.1% of female and 4.5% of male OD subjects had received VR, but no statistical difference between men and women was found when adjusting for confounding factors. Students had a higher probability of VR compared to manual workers, while the probability for agricultural entrepreneurs and nonmanual workers was lower. The use of VR was more likely in younger age groups, with the highest probability in the 18–24 and 25–34 years age groups. The probability of VR decreased as age increased.

Table 3 shows that subjects with multiple allergic ODs received VR significantly more often than those with only a single allergic diagnosis (OR 3.06, 95% CI 2.38–3.93), while subjects with other than allergic OD had a significantly lower probability of VR (OR 0.10, 95% CI 0.09–0.11).

**TABLE 3:** The Effect of Allergic Occupational Disease (OD) Diagnoses on the Probability of VR (*N* = 24,237)

No. Allergic ODs*	N	VR Cases (%)	Crude OR (95% CI)	Adjusted OR (95% CI)†
Single	3,946	1,011 (25.6)	1.00	1.00
Multiple	404	159 (39.4)	1.88 (1.52–2.33)	3.06 (2.38–3.93)
Other than allergic OD	19,887	445 (2.2)	0.07 (0.06–0.08)	0.10 (0.09–0.11)

*Allergic asthma, rhinitis, conjunctivitis, protein contact dermatitis, contact urticaria, and allergic contact dermatitis included. Irritant-induced asthma and moisture damage asthma included in the group of other than allergic OD.

†Adjusted for age group, sex, and socioeconomic group.

CI, confidence interval; OR, odds ratio; VR, vocational rehabilitation.

Table 4 presents the distribution of VR cases by field of industry. Approximately one third of all VR cases were associated with the “manufacturing” sector (513 cases), with an incidence of 1.14 cases per 10,000 person-years (95% CI 1.04–1.24). The highest incidence was observed in the “Agriculture, forestry and fishing” sector, with 1.90 cases per 10,000 person-years (95% CI 1.65–2.18), followed by “Other service activities” (including hairdressing and beauty treatment activities, etc.; 1.80, 95% CI 1.53–2.09) and “Accommodation and food service activities” (1.48, 95% CI 1.27–1.72).

**TABLE 4:** Distribution of VR by Field of Industry (ISIC Sections)

Field of Industry (ISIC Section)	*n*	VR Cases (%)	VR Incidence per 10,000 Person-Years (95% CI)	Mean Workforce (10,000)
Agriculture, forestry and fishing (A)	3,352	205 (6.1)	1.90 (1.65–2.18)	7.71
Manufacturing (C)	8,198	513 (6.3)	1.14 (1.04–1.24)	32.22
Construction (F)	3,617	137 (3.8)	0.64 (0.54–0.76)	15.29
Wholesale and retail trade; repair of motor vehicles and activities (G)	1,529	142 (9.3)	0.37 (0.31–0.44)	27.23
Accommodation and food service activities (I)	697	171 (24.5)	1.48 (1.27–1.72)	8.23
Professional, scientific and technical activities (M)	679	27 (4.0)	0.15 (0.10–0.22)	13.01
Administrative and support service activities (N)	560	25 (4.5)	0.12 (0.08–0.18)	15.06
Education (P)	531	76 (14.3)	0.34 (0.27–0.42)	16.08
Human health and social work activities (Q)	1,889	77 (4.1)	0.15 (0.12–0.18)	37.60
Other service activities (S)	583	166 (28.5)	1.80 (1.53–2.09)	6.60
Other*	2,602	76 (2.9)	0.11 (0.08–0.13)	51.06
Total	24,237	1615 (6.7)	0.50 (0.48–0.53)	230.08

*Includes fields of industry with fewer than 20 VR cases and subjects with unknown or missing information.

CI, confidence interval; VR, vocational rehabilitation.

VR utilization was also analyzed in relation to occupation. On ISCO level 3, the highest number of VR subjects was registered in “Hairdressers, Beauticians and related workers,” with a total of 173 VR subjects and an incidence of 7.20 per 10,000 person-years (95% CI 6.17–8.35). The second largest group was “Cooks,” with 147 VR subjects (incidence 2.56 per 10,000 person-years, 95% CI 2.17–3.01). The highest incidence of VR cases, at a rate of 17.40 cases per 10,000 person-years (95% CI 14.53–20.67), was observed among workers in the group “Food Processing and Related Trades Workers,” with 129 recipients, 95% of whom were bakers.

The analysis of VR use in agricultural activities was limited to ISCO level 2 due to a lack of more specific information, particularly among farmers. Within the group “Market-oriented Skilled Agricultural Workers,” 221 VR recipients were identified, with an incidence of 2.79 per 10,000 person-years (95% CI 2.44–3.19). Detailed information on VR utilization in different occupations (ISCO level 2–3) is included in the supplementary digital content (Table, SDC 2, http://links.lww.com/JOM/B954).

The primary exposure agents causing ODs leading to VR were chemicals and organic dusts. Among subjects with ODs caused by chemicals, 650 (20.7%) received VR, with resins or plastics accounting for the highest number of cases at 135 (VR proportion 23.8%). ODs due to chemicals and subsequent VR were common in manufacturing, service activities (especially beauty services), and construction. For ODs caused by organic dusts and materials, 548 (31.4%) subjects received VR. The most common subgroup linked to VR was “Flour, grains, and feeds” (275 cases, VR proportion 46.0%), with wheat accounting for nearly half of these cases (135 VR cases, VR proportion 55.8%). Among cases with ODs caused by animal-derived epithelium, hair, or excretions, 121 subjects (19.3%) received VR, with cow being the most prominent source, covering 95 of these cases (VR proportion 18.4%). Detailed information on VR usage stratified by the primary exposure agent can be found in the supplementary digital content (Table, SDC 3, http://links.lww.com/JOM/B955).

VR was typically started relatively soon after OD was recognized by the insurance company. Data on delay were unavailable for 117 cases due to missing recognition dates. VR began in the same calendar year in 39.5% of cases and in the following year for 43.2% of cases. A delay of 2 years was identified in 9.2% of cases, while a delay of three or more years was observed in 8.1% of cases, with some experiencing delays of up to 10 years.

The median duration of VR expenses was three calendar years. A 4-year duration was the most frequent, observed in 302 cases (18.7%), while 3-year (18.4%) and 2-year (18.0%) durations were nearly as common. One-year VR was implemented in 16% of cases, whereas 19.9% of cases involved VR lasting for 5 years or longer. Missing data due to ongoing VR in 2020 were identified in 182 cases (11.3%). Change in educational level or the year of degree was observed in 720 cases (50.2% of subject with finished VR) during VR or maximum 1 year later, suggesting the utilization of re-education as a rehabilitation method. An elevated post-VR educational level was observed in 304 cases (21.2%).

## DISCUSSION

We found that VR had been used in 6.7% of ODs of working-aged subjects in Finland between 2005 and 2018. Altogether 1615 subjects had received VR during the study period, averaging 115 cases every year. Our findings are in line with previous studies reported from Croatia and Canada.^6,17^

The majority of VR appeared to be linked with occupational hypersensitivity. Allergic ODs of skin (ACD and PCD/CU) together with occupational asthma and rhinitis accounted for 76.0% of all VR cases. Logically, individuals with multiple allergic ODs were more frequently provided with VR than those with a single allergic OD. This may reflect the challenge of preventing concurrent exposure via skin and airways, which could require extensive measures without VR. Research indicates that the most effective strategy for managing work-related hypersensitivity is the cessation of exposure rather than its reduction, albeit with an associated risk of adverse socioeconomic effects.^2,3,18–20^

Our data revealed that 30.1% of occupational asthma patients received VR from WCI, aligning well with the finding from a Finnish questionnaire study with 36% VR recipients (including also secondary schemes).^11^ Similarly, 26.3% of occupational rhinitis patients received VR compared to the previously reported 17% re-educated because of their OD. The difference is possibly due to the inclusion of all VR actions, not just re-education, in our study.^9^

VR can play a crucial role in protecting OD patients’ health and promoting future health and employability. Especially in the early stage of an OD, like in occupational asthma, the subject may recover after cessation of exposure and be able to achieve symptomatic recovery even without the need for medication.^21,22^ On the contrary, the continued exposure to the causative agent is usually associated with a rapid decline of lung function in occupational asthma. After the cessation of exposure, the rate of decline is slowed down, becoming similar to that observed in healthy adults.^23^ Recent results from a Finnish questionnaire study on occupational asthma patients found that two thirds had changed profession, nearly half had been unemployed after diagnosis, and more than one third had received VR because of asthma.^11^ In many professions, cessation of exposure is not possible without a change of job or an even more radical change of field of industry. A successful change from one profession to another may necessitate the use of VR. Under the Workers’ Compensation Act, the insurance company is obligated to ensure equal earning capacity for OD patients or provide compensation if the individual’s potential future employment income is diminished due to the OD. Insurance companies provide VR, including re-education up to higher education levels, to meet these legal requirements.

Different types of occupational dermatitis were common diagnoses behind VR. A Finnish long-term follow-up study on occupational hand eczema revealed that 20% of the subjects had been retrained at the expense of the insurance company after recognizing occupational dermatitis.^7^ A similar proportion of VR was found in a Canadian study on a return-to-work program for subjects with occupational dermatitis.^8^ These studies support our finding of 21.4% of ACD subjects receiving VR. However, allergic and nonallergic types of occupational dermatitis differ when examined in relation to VR. In our study, the probability of VR for PCD/CU and ACD subjects was higher than subjects with ICD, even though a previous study has reported a similar healing prognosis of allergic and nonallergic occupational eczema with or without a change of job.^24^ Documented exposure and sensitization proven by medical testing, and therefore the occupational origin of the disease, can often be confirmed in allergic skin diseases, but the causal connection between work and ICD or nonspecific dermatitis is less solid, potentially explaining the difference in VR administration. When the preferred practice after sensitization is cessation of exposure, often requiring a change of job, management of ICD may consist of the alteration of work tasks, methods, or personal protective equipment.^25^ According to the Finnish compensation policy, VR is justified mainly if ICD has become chronic and does not respond to intensified treatment and job accommodation. A Danish study showed the clearance of ICD or the improvement of symptoms after job change,^26^ indicating that a job change may be effective in managing chronic ICD.

Repetitive strain injuries are common ODs but triggered VR only rarely. Typically, strain injuries are considered as curable with appropriate ergonomic adjustments, the rotation of work tasks, and possibly a sickness absence.^27^ In some cases, these diseases continuously reoccur or cause long-term disability and a change of job may be necessary, possibly requiring VR. As a curiosity, muscular dystonia is a quite rare but known OD of musicians,^28,29^ and VR utilization has been very active according to our data.

Hand-arm vibration syndrome typically develops later in the career. After the diagnosis, minimization or cessation of exposure to hand-arm vibration is recommended, but previous studies have reported problems in both the reduction of exposure and change of job.^30^ The use of VR for hand-arm vibration syndrome appears to be relatively low, possibly due to opportunities for job accommodation or internal transfers to lower-exposure jobs within the same employer, especially in large industries.

Differences in the utilization of VR between age groups were significant even when including confounders in the regression model. The likelihood of VR diminishes along with increasing age. Due to the limited time before old age pension, the total financial impact on the insurance company could be negative toward the end of a career, as there is little time to amortize the costs of a VR investment.

Re-education appeared to be a common method of VR used to promote the future employability of OD patients. Approximately half of the rehabilitants gained a new qualification during VR, and the typical duration of VR was 3–4 years, supporting the use of re-education as part of VR. VR interventions were often initiated relatively soon after OD recognition, but about one-sixth of the rehabilitants experienced a delay of two or more years. To our knowledge, there are no studies on the impact of VR delays on return to work among OD patients. General findings suggest that early VR interventions are beneficial for a variety of health conditions, and OD is likely to be no exception.^31,32^

High molecular weight (HMW) sensitizers in the food industry and agriculture were common causative agents behind ODs leading to VR, together with a wide range of different low molecular weight chemicals in manufacturing, construction, and beauty services. A systematic review of occupational asthma recovery after exposure cessation showed a better prognosis for subjects sensitized to low molecular weight substances compared to those sensitized to HMW allergens.^22^ This underlines the importance of timely VR actions in OD patients exposed to HMW allergens. In addition, the occupation and industry where the OD patient is working should be taken into account when evaluating the need for VR. For example, exposure cessation may be impossible for a baker or farmer without a change of profession, but it may be possible by a change of tasks or the chemicals used in manufacturing. The possibilities of exposure reduction with technical measures such as local exhaust ventilation or use of personal protective equipment are possibly easier in stable settings in manufacturing than on a constantly changing construction site. In the service sector, the use of personal protective equipment may be problematic because of customer attitudes.

We found that beauty professionals relatively often received VR due to sensitization to the chemicals used. Previously, it has been shown that the career length of hairdressers is shortened because of occupational hand eczema.^33,34^ Our registry data on ODs in these professions probably does not reveal the whole picture. ODs of hairdressers and beauticians are underestimated compared to the real number of work-related diseases, because many work as self-employed entrepreneurs without WCI covering ODs.^35^ These patients face a difficult choice between possible worsening health if continuing to work or the significant socioeconomic consequences of a job change. Fortunately, these patients may be eligible for VR from secondary schemes if they do not have WCI.

### Implications for VR Provision

To maximize the likelihood of return to work, VR interventions should be implemented promptly. Only about 40% of subjects received VR within the same year that their OD was recognized. Even a delay of 1 year could be considered relatively long, but 17% of rehabilitants had a delay of two or more years. VR assessment processes should be evaluated and steps should be taken to expedite the delivery of VR services.

Although the majority of VR cases were related to allergic OD, less than one-third of subjects with allergic OD received VR. It is possible that the recommendation for exposure cessation may not always be followed. The need for VR should be carefully assessed, especially in cases of allergic ODs, and any decision to withhold VR must be well justified. Medical follow-up should be arranged if exposure reduction is chosen instead of VR.

VR use was skewed toward younger age groups, even when the analysis was adjusted for potential confounders. This suggests a potential underutilization of VR in midcareer ODs and calls for further research about effective exposure management. Continued exposure may worsen subjects’ health and increase the risk of disability. The use of VR may be warranted in more situations than it has been used to date.

### Strengths and Limitations

One strength of our study was the comprehensive registry dataset covering all ODs recognized during the study period with consistent variable coding. The registry data enabled a longitudinal evaluation of VR as a consequence of OD. Individual-level annual sociodemographic information allowed the adjustment of variables in the statistical analyses. To our knowledge, this is the first study to utilize comprehensive register data on VR utilization in ODs.

One limitation is that the register data did not offer any additional information, such as the subjects’ health and lifestyle factors, that may have influenced the rehabilitation decisions. The details of VR actions could not be directly extracted from the data, either.

The FROD contains information of ODs recognized by insurance companies. Work-related diseases of uninsured entrepreneurs are not registered, so they are not included in this study. Sociodemographic data provided by Statistics Finland represent the subjects’ situation at the end of each year, possibly causing minor errors in socioeconomic group classification.

Although our data are limited to Finland, the need for additional support for OD patients’ career continuity is universal. Especially patients with work-related sensitization probably need help in finding a new, exposure-free job or modifying their current position to limit exposure. Social security systems differ between countries, but it is crucial that the need for support is recognized at the time of diagnosis.

## CONCLUSIONS

The utilization of VR in ODs appeared to be typical, especially in allergic diseases of relatively young subjects. A significant fall in VR probability after the age of 45 years was revealed, which may indicate that there is a risk that the recommendation of exposure cessation does not materialize in older age groups. While our findings focus on the utilization of VR for ODs within the specific healthcare system in Finland, the indications of groups requiring rehabilitation after OD are likely generalizable to other countries. Further studies about VR actions taken in ODs are needed to evaluate the effectiveness of VR on employability and reducing the socioeconomic impact of ODs.
